# Behaviour of domestic rabbits during 2 weeks after weaning

**DOI:** 10.5194/aab-62-49-2019

**Published:** 2019-02-06

**Authors:** Sandra Kaźmierczak, Aleksandra Cwojdzińska, Marcin T. Górecki

**Affiliations:** Institute of Zoology, Poznań University of Life Sciences, Wojska Polskiego 71C, 60-625 Poznań, Poland

## Abstract

Thirty three rabbits from five litters that were weaned at the age of 5 weeks were observed. The animals were kept in pens that were enriched with an elevation made of bricks. In total, 150 h of observations made at feeding time (07:30–10:00 and 18:00–20:30 LT, local time) were analysed. A number of affiliative, exploratory, comfort, eating, resting and locomotor behaviours were observed. Agonistic behaviour was not observed. Rabbits showed companion and location preferences: 56 % of animals had a preferred companion, and 84 % preferred a particular place in the pen. Significant effects of group size and time of day on the frequency of some forms of behaviour were found, e.g. rabbits performed comfort behaviours more often in the morning. Sex did not influence the rabbits' behaviour. Correlations were also found between different forms of behaviour, e.g. animals that performed more exploratory behaviours also showed more locomotor behaviours and affiliative interactions.

## 1 Introduction

Rabbits are social animals and need contact with other individuals to express all forms of behaviour (Gunn and Morton, 1995). Wild rabbits live in groups comprised of one to three males and one to five females. Previous studies have compared wild and domestic rabbits, and have shown that domestic rabbits in semi-natural conditions exhibit territoriality, dominance and social behaviours that are similar to wild rabbits (Stodart and Meyers, 1964), e.g. they communicate by marking an area with buccal glands as wild rabbits do. Due to territorial behaviour, rabbits also often exhibit aggression towards one another after puberty. Castration decreases interpersonal aggression in rabbit groups (Morton, 2002).

Rabbits can be kept singly, in pairs or in groups. A rabbit kept alone is unable to interact with other individuals, and the risk of stereotypy is increased (Morisse and Maurice, 1997); however, the positive result of this kind of husbandry is that there is no aggression, and rabbits are not exposed to physical injury, e.g. bites. Keeping the animals in groups is important for adolescent rabbits. The risk of aggression is very low (before puberty), and rabbits can play together and display the entire spectrum of social behaviour. Studies show that at a density of more than 16 rabbits/m${}^{2}$, the welfare of rabbits decreases due to overcrowding (Morisse and Maurice, 1997). Therefore, providing enough space is important due to the risk of overcrowding. It is known that less “moving space” is available for rabbits kept in small cages (e.g. due to the hight of the cage restricting mobility) than in larger cages with a higher animal density. Hence, cages should be (at least) 75–80 cm long and 35–40 cm wide to allow animals to carry out natural behaviours (EFSA, 2005). Rabbits held at a density that is too high exhibit less social and locomotor behaviours and more behaviours related to grooming and exploration (Morisse and Maurice, 1997).

Weaning is a stressful situation for all domestic animals. Weaned rabbits should be kept in litter groups or in mixed groups of animals of the same age (Hawkins et al., 2008). In intensive breeding, rabbits are weaned at the age of 21 days (Hoy et al., 2006). Rabbits should be weaned at 4–6 weeks of age. It is ideal to transfer the mother to another cage and leave the kits in the original cage (where they lived until weaning), although this is rarely applied under breeding conditions. Kits can be kept together until the age of 3 months, or until they reach puberty (Fournier, 2008).

**Table 1:** Parents of the rabbits observed, the litter size and the sex ratio.

Litter no.	Father's breed	Mother's breed	Litter	Sex ratio
			size	()
1	Termond White	Termond White	5	1:4
2	Termond White	Termond White	6	4:2
3	Termond White	Silver Marten × New Zealand White	9	5:4
4	Termond White	Silver Marten × New Zealand White	4	2:2
5	Termond White	Termond White × Silver Marten × New Zealand White	9	5:4

Social stimulation is very important in rabbits owing to their social nature. Studies in semi-natural systems indicate the occurrence of a hierarchy between rabbits and behaviours such as allogrooming, lying and eating in the company of another individual (Held et al., 1995). Enriching the social life of rabbits by placing at least two individuals together introduces more benefits than the use of objects to diversify the environment. This also prevents the occurrence of stereotypy as it introduces more unforeseen situations due to the presence of another individual (Chu et al., 2004). The role of environmental enrichment in captive rabbits' lives is enormous. Studies have shown that rabbits kept in cages with chewing sticks, shelters where they can hide and/or elevations show less stereotypy, more social behaviour and less aggression toward other animals in the group (Princz et al., 2007; Zucca et al., 2012).

Previous studies have also reported that the housing system (Trocino et al., 2008), the group and cage size, and the density (Chu et al., 2004; Princz et al., 2008; Buijs et al., 2011a) influence the behaviour of growing rabbits.

The aim of this study was to characterize behaviours expressed by young rabbits after weaning and to determine the occurrence of company and/or location preferences. Moreover, the potential influence of factors such as sex, body weight, group size, and time of day/week of observation on the frequency of observed behaviours was investigated.

## 2 Materials and methods

### 2.1 Subjects and pens

The observations took place in July/August 2013 at the “Zielona Chata” agro-tourism farm in Cichowa, Wielkopolska Province, Poland.

A total of 33 rabbits (16 females and 17 males) from five litters were studied. There were nine animals in two litters and four, five and six animals in other three respective litters. During observations two individuals died. One of the animals (from a litter consisting of six siblings) died early in the experiment and was not included in further analysis. In spite of the death of rabbit no. 8 from the fifth group a day before the final weighting, the observations of its behaviour were included in this paper. Thus, data from 32 individuals were analysed. The litters were from five females that were crossed with two males (Table 1). Each litter was placed in a purpose-built enclosure. Each pen was the same size – 0.85 m (length) × 0.65 m (width) – and contained identical sets of bowls with food and water, hay as bedding and an elevation (made of two bricks and a paving stone) to enrich the environment. The five pens were placed in one building. Rabbits were given pellet rabbit food at 07:30 and 18:00, and water was always available. The rabbits were weaned at the age of 5 weeks. Each individual was marked with animal marker and weighed at beginning and the end of the observations.

### 2.2 Observations

**Figure 1:**
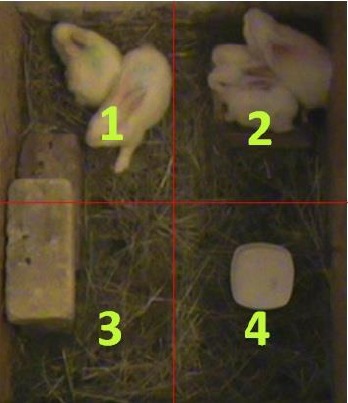
The pen divided into four virtual squares.

Observations began the day after weaning to get the rabbits acquainted with the new environment. Observations were carried out over 2 weeks at the end of July and beginning of August 2013. Each group was observed from 07:30 to 10:00 am and from 18:00 to 20:30 pm, 6 days a week. In total, 150 h of observations were made by one observer (A.C.). The continuous recording method and the sampling of all occurrences of behaviours method were used (Altmann, 1974). Observations of groups 3 and 4 were carried out directly on site, while the rest of the observations (groups 1, 2 and 5) were noted down from video recordings. Both the behaviour and the position of the rabbits were recorded. An ethogram was developed based on previous ethograms used (Morton et al., 1993; Gunn and Morton, 1995; Hawkins et al., 2008; Jordan et al., 2010). The pens were split into four virtual squares in order to calculate location preference (Fig. 1). In square number 2 there was a bowl of food, the elevation was located in square number 3 (and partially in square 1), and a bowl of water was in square number 4. Every 30 min the position of each individual was noted. The observation stand was near squares number 3 and 4.

### 2.3 Statistics

The data were recorded and analysed using SPSS 20.0 (for Windows), and each category of behaviour was analysed using the Pearson correlation coefficient and a univariate ANOVA (sex and litter size). A paired t test was carried out to estimate the differences in behaviour between the time of day and the week of the experiment. Companion preference was calculated on the basis of the frequency of the occurrence of the behaviour according to the following formula:

1 $$Z=\frac{\mathrm{observed}\;\mathrm{frequency}-\mathrm{expected}\;\mathrm{frequency}}{\sqrt{\mathrm{expected}\;\mathrm{frequency}}}$$

Each individual with Z > 1.96 was considered preferred, whereas Z < -1.96 meant that an individual was avoided (Van Dierendonck et al., 2009).

Location preference was analysed using a Chi square test.

## 3 Results

**Figure 2:**
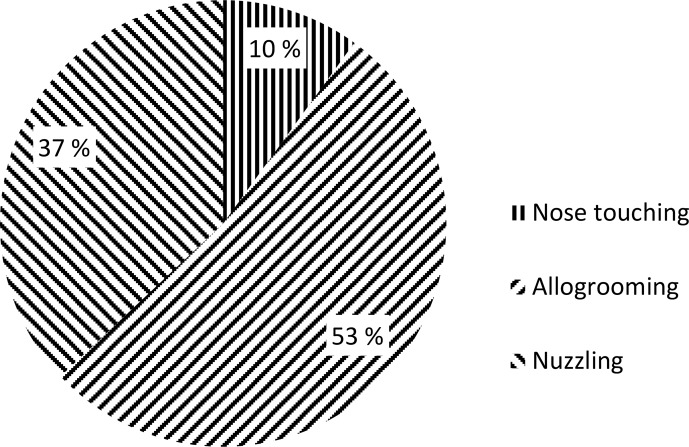
Frequency of affiliative interactions (as a percentage).

**Figure 3:**
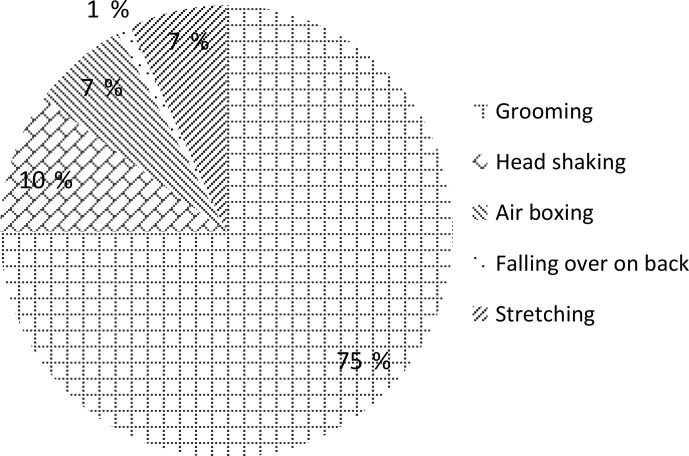
Frequency of comfort behaviours (as a percentage).

**Figure 4:**
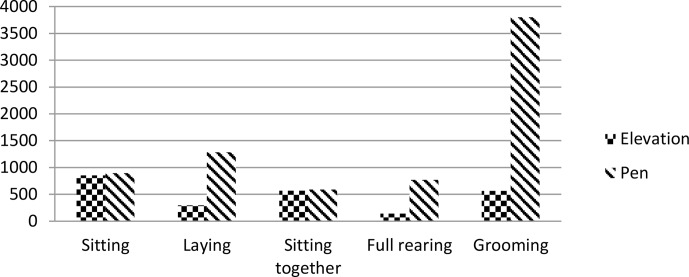
Numbers of behaviours observed on the elevation and in other parts of the pen.

The classification and descriptive information regarding the observed forms of behaviour are shown in Table 2. Vocalization, agonistic behaviours, chin marking, thumping and sexual behaviour were not observed. Fights while eating and drinking or attempts to defend food bowls were not observed. Rabbits did not chase other animals away, and they were not aggressive toward one another (no signs of biting, etc.). Defecation was not reported due to the difficulty involved with observing this behaviour. The most frequent affiliative behaviour involving another animal was allogrooming, and the least frequent affiliative behaviour was nose touching (Fig. 2). The most frequent comfort behaviour was grooming (Fig. 3). The most frequent behaviour on the elevation was sitting. All forms of behaviour observed on the elevation were also recorded in other parts of the pen (Fig. 4).

**Table 2:** Forms of behaviour observed in rabbits.

Behaviour	$\bar{x}$	SD
Comfort behaviour		
Grooming	118.8	40.3
Air boxing	12	7.9
Falling down on back	1.8	3.2
Head shaking	17.9	11.4
Stretching	12.3	6
Eating behaviour		
Eating hay	46.9	21.3
Eating pellets	84.5	20.5
Drinking water alone	50	28.8
Drinking water accompanied by another animal	17.1	6.5
Resting behaviour		
Sitting alone	27.9	11.7
Sitting accompanied by another animal	18.5	15.5
Laying alone	40.2	27.4
Laying accompanied by another animal	77.4	24.1
Drowsing alone	3.6	3.4
Drowsing accompanied by another animal	9.5	5.9
Locomotor behaviour		
Running	14	9.4
Jumping	106.6	51
Capering	10.2	7.6
Trying to escape the pen	2.2	3.3
Exploratory behaviour		
Rearing	3.6	3.0
Full rearing	24.1	14.5
Leap rearing	10.3	7.7
Sitting alert	4.6	5.9
Sniffing	9.7	6.1
Paw scraping	7.5	7.5
Affiliate behaviour with another animal		
Allogrooming	17.6	16.1
Nuzzling	12.4	7.3
Nose touching	3.6	2.3
Returned nose touching	3.6	2.4
Behaviours performed on the elevation		
Sitting	26.6	16.1
Laying down	9.2	11.1
Full rearing	4.4	5.1
Sitting on the brick half step	9.4	7.9

### 3.1 Companion preference

Some rabbits in each group showed a companion preference (four out of five individuals in the first group, and two of five, four of nine, two of four and six of nine in the other respective groups). These individuals preferred the company of one particular rabbit (only two rabbits had two preferred siblings). Twelve out of twenty-one preferences were reciprocal. No specific traits that could have affected the companion preference were found. Some rabbits avoided the company of particular siblings (all five rabbits in the first group and two of five, two of nine, one of four and one of nine in the other groups, respectively). Each of these animals avoided only one sibling. Four of eleven avoidance relationships were reciprocal.

**Table 3:** Number of animals that displayed location preferences.

	Number of animals that preferred virtual square				
Group	Virtual	Virtual	Virtual	Virtual	No preference
	square	square	square	square	
	no. 1	no. 2	no. 3	no. 4	
1	0	5	0	0	0
2	0	5	0	0	0
3	3	3	2	0	1
4	1	3	0	0	0
5	2	3	0	0	4

### 3.2 Location preference

The majority of rabbits exhibited a location preference. Table 3 presents the number of animals that showed a location preference in detail.

A preference for virtual square number 2 was seen in every group, specifically in groups number 1 and 2. Rabbits in these groups notably rested in the food bowl, while rabbits in the other groups preferred to relax in virtual square number 1. Rabbits from group 3 gladly used the elevation.

### 3.3 Correlations between observed behavioural categories

**Figure 5:**
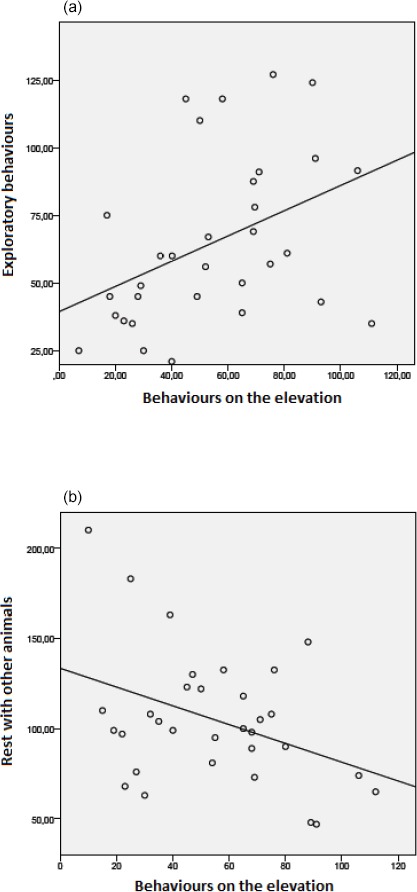
Correlations between **(a)** behaviours performed on the elevation and exploratory behaviours (n=32, r=0.42, p < 0.05), and **(b)** behaviours performed on the elevation and rest with other animals (n=32, r=-0.41, p < 0.05).

Rabbits that displayed more behaviours on the elevation showed more exploratory behaviours (Fig. 5a; n=32, r=0.42; p < 0.05), but they showed fewer behaviours related to rest with other animals (Fig. 5b; n=32, r=-0.41; p < 0.05).

**Figure 6:**
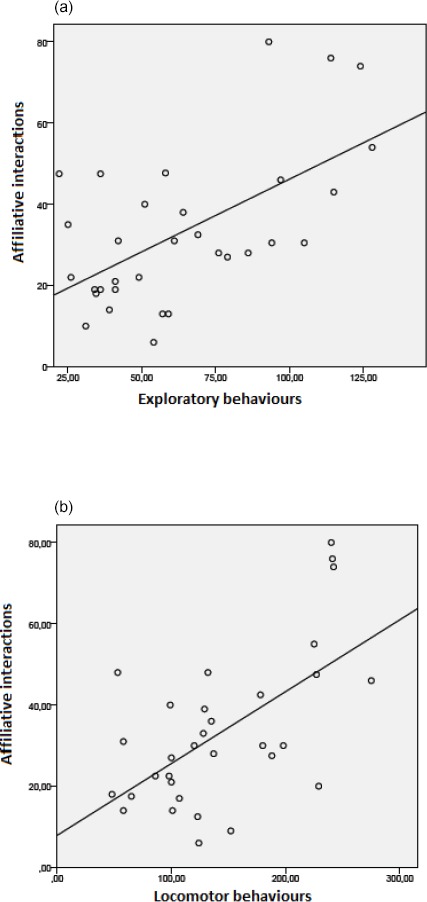
Correlations between **(a)** affiliative interactions and exploratory behaviours (n=32, r=0.59, p < 0.01), and  **(b)** affiliative interactions and locomotor behaviours (n=32, r=0.6, p < 0.01).

**Figure 7:**
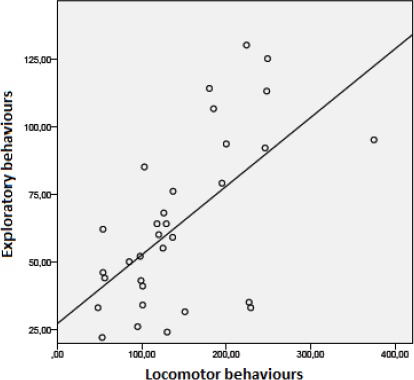
The correlation between locomotor and exploratory behaviours (n=32, r=0.64, p < 0.01).

Individuals that displayed more exploratory behaviours also showed more affiliative behaviours directed towards other animals (Fig. 6a; n=32, r=0.59; p < 0.01). More active rabbits performed more affiliative behaviours with other animals (Fig. 6b; n=32, r=0.6; p < 0.01) and more exploratory behaviours (Fig. 7; n=32, r=0.64; p < 0.01).

**Figure 8:**
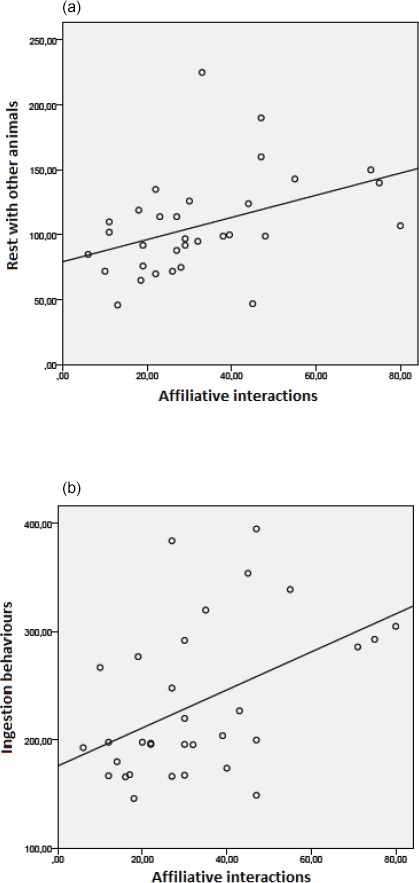
Correlations between **(a)** affiliative interactions and rest with other animals (n=32, r=0.43, p < 0.05), and **(b)** affiliative interactions and ingestion behaviours (n=32, r=0.46, p < 0.01).

Animals that showed more affiliative interactions with other rabbits also rested with other animals more often (Fig. 8a; n=32, r=0.43; p < 0.05) and performed more eating behaviours (Fig. 8b; n=32, r=0.46; p < 0.01).

**Figure 9:**
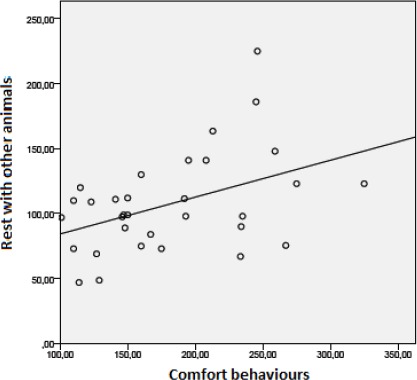
The correlation between comfort behaviours and rest with other animals (n=32, r=0.44, p < 0.05).

Rabbits that performed more comfort behaviours rested with other individuals more often (Fig. 9; n=32, r=0.44; p < 0.05).

### 3.4 The influence of sex

The analysis did not show a significant impact of sex on the frequency of any form of behaviour (p > 0.05).

### 3.5 The influence of weight

Weight is linked to genotype. The heaviest animals were 1/2 Termond White, and the lightest were 3/4 Termond White. Analysis showed that smaller individuals sat (with other animals) on the elevation (r=-0.35; p < 0.05) and lay in the food bowl (r=-0.1; p < 0.01).

### 3.6 The influence of group size

**Figure 10:**
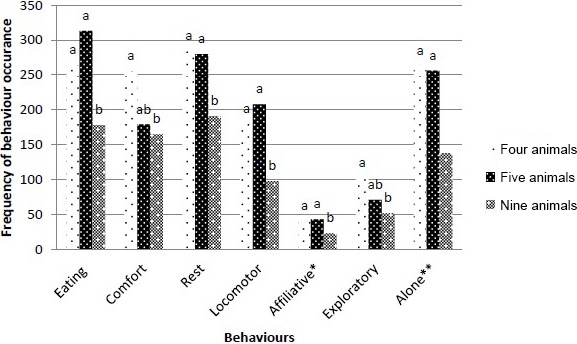
The influence of group size on the frequency of the occurrence of behaviours. Differences between the means indicated by the letters “a” and “b” are significant (p < 0.05). ${}^{*}$ Interactions with another animal. ${}^{**}$ Laying, sitting, dozing, or eating and drinking alone.

A one-way ANOVA showed that less affiliative behaviours, eating behaviours and resting behaviours were observed in groups of nine animals compared with groups comprised of four or five animals; furthermore, less comfort behaviours and exploratory behaviours were noted in groups of nine animals compared with groups of four animals (Fig. 10). Litter size did not impact the occurrence of behaviours on the elevation.

### 3.7 Week of observation

The analysis did not a show significant impact of the observation week on the frequency of any form of behaviour (p > 0.05) due to the rabbits' young age (before puberty).

### 3.8 Time of day

The t test results showed that comfort and rest behaviours (especially with other animals) were often performed in the morning, while eating behaviour occurred more often in the evening (Table 4).

## 4 Discussion

A properly diversified living space for growing rabbits kept in small groups has a positive effect on their welfare. In this particular study, individuals that exhibited more exploratory behaviours also displayed more behaviours on the elevation. This suggests that rabbits used the elevation to more effectively explore their environment. The negative correlation between behaviour performed on the elevation and rest with other animals (Fig. 5b) could suggest that being on the elevation prevented group leisure (due to the small area available) or that individuals preferred resting together somewhere other than on the elevation (e.g. near the food bowl).

**Table 4:** Differences in the occurrence of behaviours with respect to the time of day: morning vs. evening (${}^{*}p$ < 0.05).

Category of behaviour	t	Significance
Comfort behaviours	3.066	0.04${}^{*}$
Resting	4.098	0.02${}^{*}$
Activity	-1.068	0.35
Exploration	2.678	0.06
Affiliative behaviour	-0.373	0.73
Behaviours performed on the elevation	1.829	0.14
Eating	-4.947	0.01*
Resting alone	-0.638	0.56
Resting with other animals	3.394	0.03${}^{*}$

Animal density is more important in rabbit husbandry than the use of the appropriate bedding (Matics et al., 2003). In this study there were significantly less social, exploratory, ingestion, comfort, locomotory and resting behaviours in groups of nine individuals (compared with smaller group sizes). This may have been caused by an animal density that was too high. In the experimental pen designed for this study (0.55 m${}^{2})$, an animal density of 8.8 individuals/m${}^{2}$ would have been appropriate according to guidelines (https://www.pzhk.pl/wp-content/uploads/Rozporzadzenie_Dz.U.2010.116.778.pdf, last access: 20 April 2014); however, according to body weight guidelines a total of 22 kg in each pen would have been suitable. Therefore, owing to the young age and low body weight of the animals used, the pen was an appropriate size and there was no overcrowding. Morisse and Maurice (1997) showed that rabbits kept at a density that is too high exhibit less social and locomotory behaviours and more comfort and exploratory behaviours. At a density of over 20 rabbits/m${}^{2}$, the animals' rest time increases, feeding time shortens and less activity is performed. Trocino and Xiccato (2006) observed more comfort and exploratory behaviours and no stereotypy in rabbits bred in groups, compared with animals that were kept alone. This indicates that keeping rabbits in groups is beneficial for their welfare. In a more recent study, Trocino et al. (2008) showed no significant differences in behaviour when comparing a 12 rabbit/m${}^{2}$ group with a 16 rabbits/m${}^{2}$ group. Therefore, this confirms the optimal density of rabbits put forward in the 2005 EFSA report. An incorrectly chosen density, which is either too low or too high, can adversely affect rabbit growth. Consequently, the size of the cage and the number of animals housed should be optimized (Trocino et al., 2008).

The number of animals in a group can influence rabbit behaviour. In smaller groups rabbits rest more often than in bigger groups. More locomotor behaviours have also been observed in groups of more than 15 individuals (Princz et al., 2008). Furthermore, rabbits in bigger groups consume less food. When the number of rabbits exceeds seven, feed conversion efficiency is reduced. Studies confirm that it is important to select a sufficiently large group and to keep rabbits at the correct density, as specified by the standards, so that rabbit breeding is as effective as possible without harm to the animals. Breeding rabbits in large groups has more disadvantages (bodily injury, a higher risk of disease and aggression, and lower meat quality) than it has advantages (social behaviour and a larger area). The best conditions for rabbits are groups of 4–5 individuals and a density of 15–17 rabbits/m${}^{2}$ (Szendrö and Zotte, 2011).

Ingestion and rest behaviours are often performed in the company of another individual. This displays the social bond between rabbits. In this study, animals were close to each other while lying down, sitting, drowsing, drinking and eating. The fact that these behaviours are performed together has also been evidenced by previous studies such as Held et al. (1995) and Zucca et al. (2012). Locomotory and exploratory behaviours are more often performed alone. Jordan et al. (2010) showed that rabbits prefer to eat and drink in the company of other individuals. Under these conditions, they ingest smaller amounts more often instead of long periods of grazing. During this research, rabbits also stayed together on the elevation; however, this was correlated with the rabbits' weights (smaller individuals were observed on the elevation more often with another individual).

According to our results, rabbits showed a companion preference for an individual with whom they performed the above-mentioned activities. Rabbits weaned at a young age (21 days) tend to stay in groups. Matics et al. (2004) observed (in his free choice system) that rabbits are more likely to choose the company of other individuals than to be alone after weaning: rabbits chose small cages, where the density reached 50–70 rabbits/m${}^{2}$. A study conducted by Zotte et al. (2009) indicated that when rabbits were given a choice between a cage with a mirror or one without, 72 % of the animals chose the cage with a mirror. Keeping rabbits in wired mesh cages allows them contact with other individuals. Hence, it is important to provide animals with the opportunity to express social behaviour in rabbit husbandry (Szendrö and McNitt, 2012).

No agonistic behaviours were observed in this study. Vervaecke et al. (2010) showed that rabbits form a linear hierarchy at the age of 10 weeks. The fact that observations ended at the age of 7 weeks explains the lack of agonistic behaviours, which made it impossible to establish a hierarchy in the groups of rabbits studied. Rabbits should be slaughtered before reaching 80 days (before sexual maturity) due to increased aggression (Trocino and Xiccato, 2006). Aggression is also correlated with the number of individuals kept: the more rabbits, the higher the probability of aggressive behaviour (Szendrö et al., 2009). The results of a study by Trocino and Xiccato (2006) showed that the occurrence of aggressive acts increases with the age and the size of the group.

The correlations between exploratory behaviours, affiliative interactions and locomotory behaviours were highly significant in this research. More active individuals were more likely to exhibit social behaviours, which is in agreement with the above-mentioned results published by Trocino and Xiccato (2006): rabbits with the ability to exhibit locomotor behaviours (in a suitable cage or enclosure) displayed more inter-individual contact behaviours. It was observed that comfort behaviour was correlated with resting in the company of another individual. During this research, it was possible to observe the frequent occurrence of both behaviours alternately. Jordan et al. (2010) found that grooming is the most common comfort behaviour. The high incidence of comfort behaviours may also be related to the bedding used. Clean fur is important for rabbits, so they groom it often. Morisse et al. (1999) showed that the dirtier the deep bedding becomes, the more often rabbits exhibit grooming.

The impact of sex on the occurrence of behaviours was not observed due to the young age of the rabbits. It is said that behavioural differences occur after puberty (Morisse and Maurice, 1997).

The rabbits studied most often stayed in the second virtual square of the pen. These results probably arise from the fact that rabbits were eating during the observation periods (time of feeding), and that the bowls were placed in virtual square number 2. However, the location preference observed may also be related to the fact that rabbits chose resting places near the eating site (Negretti et al., 2004). This fact also explains the preference for the first virtual square shown by some individuals. The preference for the first and second squares could also be explained by the tendency of rabbits to select places near walls to rest. Rabbits are less likely to rest in central areas. Buijs et al. (2011b) reported a high occurrence of rabbits resting near the walls, and proposed that rabbits do not fear attack from predators in such places. The rabbits in this study also often rested beside the elevation. The introduction of the brick construction (the elevation) meant that rabbits were often observed next to it and not just near the walls. In all groups, animals defecation was mainly observed in the corner of virtual square 3, next to the elevation. The selection of a specific place for excretions is also observed in the wild. Wild rabbits leave their droppings outside the burrow, near its entrance, often near a molehill if there is one close to the burrow (Denenberg et al., 1969).

Rabbits are considered to be nocturnal or crepuscular animals. Studies on their daily activity show that resting behaviours most frequently occur during the day and that all activities (locomotory, exploratory, comfort) occur at night (Gunn and Morton, 1995; Piccione et al., 2007; Jordan et al., 2010; Buijs et al., 2011a; Gianetto et al., 2016). The observed rabbits mostly showed eating behaviour in the evening. This may be related to observation time, as the evening observation period was closer to sunset than the morning observation period was to sunrise. Domestic rabbits eat in the evening and at night, as do wild rabbits. Feeding in the evening reduces the occurrence of stereotypy in rabbits (Krohn et al., 1999). Buijs et al. (2011a) observed that rabbits consumed most of their food during the period around sunset.

This study has brought us to several conclusions. Time of day and litter size influenced some behaviours. Rabbits displayed more ingestion behaviours in the evening and more comfort and resting behaviours (especially with other individuals) in the morning. The highest number of behaviours was observed in groups of four or five individuals; in bigger groups (e.g. nine individuals) the frequency of behaviours decreased. There was no significant effect of sex on the frequency of the behaviours observed. Weaned rabbits did not show agonistic behaviour due to their young age (before puberty). Rabbits (in all groups) exhibited companion and location preferences. The factor/s influencing the choice of companion was not determined. Different forms of behaviour were related, e.g. the correlations between exploratory and affiliative behaviours with other individual and locomotor behaviours were highly significant.

Further studies such as this one could provide more important information on behaviour in weaned rabbits.

## Data Availability

The data sets are available upon request from the corresponding author.
